# A Prospective Cohort Study of COVID-19: Evaluation of the Early Role of IL-1 and IL-6 Antagonists in Improving the Outcome of the Illness and Reduction in the Risk of Death

**DOI:** 10.3390/healthcare11071025

**Published:** 2023-04-03

**Authors:** Mardheya Al. Kharusi, Naffesa Al Sheikh, Maiya Alhajri, Seif Ali Al. Mandhri, El-Sayed Khafagy, Ehssan H. Moglad, Hadil Faris Alotaibi, Wael A. H. Hegazy

**Affiliations:** 1 Pharmacy Department, Field Hospital, Muscat 111, Oman; 2 Department of Pharmaceutics, College of Pharmacy, Prince Sattam Bin Abdulaziz University, Al-kharj 11942, Saudi Arabia; e.khafagy@psau.edu.sa (E.-S.K.); e.moglad@psau.edu.sa (E.H.M.); 3 Department of Pharmaceutics and Industrial Pharmacy, Faculty of Pharmacy, Suez Canal University, Ismailia 41522, Egypt; 4 Department of Microbiology and Parasitology, Medicinal and Aromatic Plants Research Institute, National Center for Research, Khartoum 2404, Sudan; 5 Department of Pharmaceutical Sciences, College of Pharmacy, Princess Nourah bint Abdulrahman University, Riyadh 11671, Saudi Arabia; hfalotaibi@pnu.edu.sa; 6 Department of Microbiology and Immunology, Faculty of Pharmacy, Zagazig University, Zagazig 44519, Egypt; 7 Pharmacy Program, Department of Pharmaceutical Sciences, Oman College of Health Sciences, Muscat 113, Oman

**Keywords:** COVID-19, COVID-19 treatment, Interleukins, IL-1 antagonist, IL-6 antagonist, anakinra, tocilizumab

## Abstract

The COVID-19 pandemic had a profound impact on global health, economies, and social systems. The crucial factor that determines the success of COVID-19 treatments is preventing the need for mechanical ventilation and intensive care admission. In the context of COVID-19, several treatments have been found to play a role in the disease’s progression and severity. Interleukins (ILs) have been identified as key mediators of the cytokine storm that can occur in severe cases of COVID-19, leading to respiratory failure and other complications. For instance, IL-1 antagonist (anakinra) and IL-6 antagonist (tocilizumab) are supposed to be promising treatments as well as cortisones for COVID-19. This prospective study aims to evaluate the effectiveness of anakinra or tocilizumab in addition to cortisone in preventing the progression of mild to moderate COVID-19 cases to severe intensive care admission. Biochemical and hematological parameters, such as D-dimer, ferritin, LDH, CRP, and white blood cells (WBCs), were measured after treatment with either anakinra or tocilizumab in addition to cortisone or cortisone alone. The study also recorded the number of deaths and patients admitted to intensive care. The results indicate that anakinra significantly improved outcomes and decreased the number of intensive care admissions compared to tocilizumab or cortisone alone. Therefore, anakinra may play a vital role in controlling the progression of COVID-19, and its use in mild to moderate cases may prevent the worsening of the disease to severe stages.

## 1. Introduction

The Coronavirus disease 2019 (COVID-19) is caused by the severe acute respiratory syndrome coronavirus 2 (SARS-CoV-2), which is a type of single-stranded RNA virus in the family *Coronaviridae* [1,2]. The COVID-19 pandemic, caused by the novel coronavirus SARS-CoV-2, has affected the world in an unprecedented manner. The virus has led to a massive surge in global morbidity and mortality, with over 150 million cases and over 3 million deaths reported as of early 2023 [3,4,5]. The virus primarily infects the respiratory tract and causes a wide range of symptoms, ranging from mild cold-like symptoms to severe pneumonia and multi-organ failure [6,7,8,9]. The pathogenesis of COVID-19 is complex and not fully understood, but it is believed that the virus enters the body through the ACE2 receptors, which are abundant in the respiratory tract, and causes an immune response that leads to inflammation and tissue damage [6,10,11,12]. The complications of COVID-19 can be severe and long-lasting, ranging from acute respiratory distress syndrome (ARDS) to multi-organ failure, thromboembolism, and even death [6,7,8,11,13]. Furthermore, COVID-19 has been shown to cause long-term damage to the respiratory, cardiovascular, and nervous systems, leading to persistent symptoms even after recovery from the acute phase of the illness [7,8,14,15,16,17].

The worst-case scenario of the prognosis of COVID-19 is the development of a cytokine storm, a phenomenon that occurs in some severe cases of COVID-19, where an excessive and uncontrolled release of cytokines leads to a hyperinflammatory response in the body [18,19,20]. This overactive immune response can cause significant damage to various organs and tissues, leading to severe symptoms and, in some cases, death [20,21,22,23,24,25]. The virus triggers the release of cytokines, which signals the immune system to respond and fight off the infection. However, in some cases, the immune response becomes excessive, leading to a cytokine storm. This results in a widespread and uncontrolled inflammation that can cause damage to the lungs, heart, and other organs [20,26,27]. Symptoms of a cytokine storm can include fever, fatigue, shortness of breath, and multiple organ failure. It is also associated with increased severity of illness and a higher risk of death [19,20,21]. The exact mechanisms underlying the development of cytokine storm in COVID-19 are not well understood, but it is thought to involve a complex interplay between the virus, the host immune response, and genetic and environmental factors [20,22,27,28]. This immune response is characterized by the overproduction of cytokines, particularly Interleukins IL-1 and IL-6, which play a crucial role in the development of severe COVID-19 [27,29,30,31,32,33,34]. Treatment of cytokine storm in COVID-19 involves controlling the hyperinflammatory response, which can be achieved through the use of drugs that block cytokines or modulate the immune response. Some examples of these drugs include corticosteroids, monoclonal antibodies, and interleukin antagonists [35,36,37,38].

IL-1 and IL-6 are among the cytokines that play crucial roles in regulating the cellular immune response to intracellular infections such as viral infections [20,26,31,39]. IL-1 is a proinflammatory cytokine that plays an important role in the immune system’s response to infection and tissue injury. It is produced by a variety of immune cells, including macrophages, monocytes, and dendritic cells, and acts to recruit and activate other immune cells [30,40,41]. IL-1 has been implicated in the pathogenesis of many inflammatory and autoimmune diseases, including rheumatoid arthritis, gout, and systemic juvenile idiopathic arthritis [40,42,43]. IL-6 is involved in a wide range of immune responses in the body and is produced by a variety of immune cells, including T cells, B cells, macrophages, and fibroblasts [31,42]. Elevated levels of both IL-1 and IL-6 have been observed in individuals with severe cases of COVID-19 and are thought to contribute to the hyperinflammatory response seen in these patients [29,31,37,44]. As a result, blocking the activity of IL-1 and IL-6 has been proposed as a potential therapeutic strategy for the treatment of COVID-19 [35,38,45]. IL-1 receptor antagonist, anakinra, has been used to treat a variety of inflammatory disorders, including rheumatoid arthritis and psoriasis, and is being tested for its potential to treat COVID-19 [30,38,40,42]. IL-6 antagonist, tocilizumab, is a monoclonal antibody that binds to and neutralizes IL-6 [42,46]. In clinical trials, tocilizumab and anakinra have shown promise in reducing the severity of symptoms and improving outcomes in COVID-19 patients, and have been associated with a reduction in the need for mechanical ventilation and improved survival rates in patients with severe COVID-19 [30,31,35,38,40,42,46].

The treatments for COVID-19 have been limited and mostly supportive, but there has been growing interest in the use of IL-1 and IL-6 antagonists in the treatment of COVID-19 to reduce the severity of symptoms, improve the outcome of the illness, and potentially reduce the risk of death [35,42]. However, IL-1 and IL-6 antagonists have potential adverse effects that have been reported with their use in COVID-19 treatment [47]. As immune suppressors, IL-1 and IL-6 antagonists could increase the risk of infections, particularly opportunistic infections. In addition to expected allergic reactions, IL-1 and IL-6 antagonists could increase the risk of thrombosis and have been associated with increased gastrointestinal symptoms such as diarrhea, nausea, vomiting and liver toxicity [48,49,50,51]. The aim of the current prospective cohort study in this context is to assess how the severity of symptoms and the outcome of COVID-19 can be controlled and improved when IL-1 antagonist anakinra and IL-6 antagonist tocilizumab are prescribed early to individuals with mild to moderate cases. There are several studies which evaluated the effectiveness of anakinra and tocilizumab in treatment of COVID-19 [33,34,52,53,54,55,56,57,58,59]; however, there are no studies which evaluated their roles in controlling COVID-19 when given in early stages of infection, as far as we know. The effects of anakinra and tocilizumab on the levels of some biomarkers and illness outcomes were assessed in mild to moderate COVID-19 cases.

## 2. Materials and Methods

### 2.1. Participants

A cohort, prospective, multicenter, national, analytical study was conducted employing the data from patients admitted in Muscat hospital suffering from mild to moderate COVID-19 symptoms. Enrollment began from 1 June and continued to 30 December 2022.

### 2.2. Inclusion Criteria

All admitted patients who were diagnosed with COVID-19, suffering from mild to moderate signs, were considered in the prospective study. The mild to moderate cases were defined as those patients who were suffering from fatigue, fever, dyspnea, loss of taste, loss of smell, respiratory distress, cough, sore throat, diarrhea, abdominal pain and vomiting, and were clinically diagnosed and PCR-confirmed as COVID-19. The data collection was restricted to patients who had not received the COVID-19 vaccine or suffered from COVID-19 infection in the 6 months before starting the prospective study.

### 2.3. Exclusion Criteria

The data of admitted patients who were suffering from severe illness including low oxygen saturation (SpO_2_ < 95%) and/or difficulty of breathing were omitted. All the deteriorated cases that were transferred to intensive care (ICU) or died within one week after administration of treatment were omitted. The data of asplenic patients, those with autoimmune-disease, cancer patients and children below 12 years of age were not counted.

### 2.4. Study Design

The data were collected from patients who were administrated the usual prescribed COVID-19 treatment regimen according to national guidelines and were also treated with IL-1 antagonist (anakinra), IL-6 antagonist (tocilizumab), or not. The patients were distributed randomly in three groups according to the treatment regimen as illustrated in Table 1. The independent variables that are hypothesized to cause an effect are assumed to be anakinra or tocilizumab while the dependent variables are as biochemical and hematological parameters during treatment of mild to moderate COVID-19 patients. The levels of biochemical parameters as levels of ferritin, D-dimer, C-reactive protein (CRP), Lactate dehydrogenase (LDH), and hematological parameters including white blood cells (WBCs) and neutrophils counts, were compared before and after treatment to evaluate the outcome of the illness. Furthermore, the clinical improvement was assessed as were the numbers of patients who were transferred to the ICU or died at the end of treatment in the three groups.

### 2.5. Statistical Analysis

The statistical significance between the levels of the measured biochemical or hematological parameters was assessed using a paired t-test or a Wilcoxon signed-rank test as appropriate. A one way ANOVA test was employed to assess the statistical significance of the changes in the parameter levels before and after treatment in the three tested groups. The patients who were transferred to ICU or died were counted over five weeks after finishing the treatment and plotted using the Kaplan–Meier method, and the Log-rank test was employed to attest the statistical significance. A chi-square test was used to compare the difference in the proportion of numbers of deaths and ICU-admitted patients among the three tested groups, followed by a Bonferroni post-hoc test to detect significant differences between groups. The statistical analysis was carried out using GraphPad Prism, and *p* < 0.05 was considered statistically significant.

### 2.6. Ethical Approval

Our study used information from the Omani Ministry of Health database through the Al Shifa platform. All the used data were analyzed after removing all identifiers. The current study was approved by the Center of Studies and Research, Directorate General of Planning and Studies, Ministry of Health, Sultanate of Oman (MOH/CSR/22/26257).

## 3. Results

A total of 275 patients who were recruited in the current study were divided into three groups (Table 2). The first group who received the usual treatment regimen (80 cases) were considered the control group. The second and third groups who additionally received IL-1 or IL-6 antagonists comprised 95 and 100 cases, respectively.

### 3.1. The Usual Regimen Did Not Improve the Biochemical Parameters Significantly

**Figure 1:**
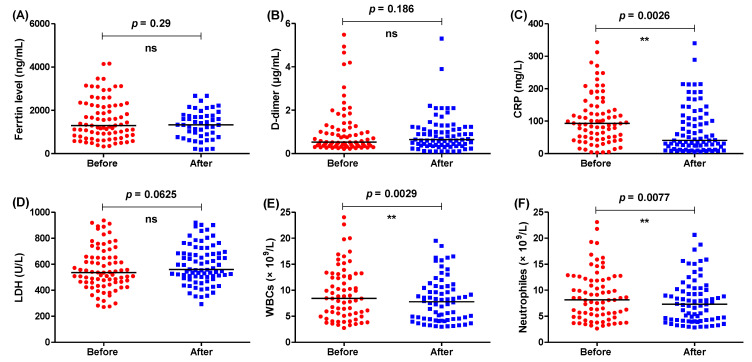
The levels of different parameters before and after administration of treatment regimen without interleukin antagonists. The levels of (**A**) ferritin, (**B**) D-dimer, (**C**) CRP, (**D**) LDH, (**E**) WBCs, and (**F**) neutrophiles were measured before and after completion of treatment regimen, ** = *p* < 0.01, and ns (nonsignificant) *p* > 0.05.

The levels of biochemical and hematological parameters were measured before and after administration of the usual treatment regimen (Figure 1). The numbers of WBCs and neutrophils were significantly decreased after completion of the doses; however, there was no significant difference between the levels of ferritin, D-dimer and LDH before and after treatment. The levels of CRP were significantly lessened after completion of the course of treatment, perhaps due to the dexamethasone’s anti-inflammatory effects.

### 3.2. Anakinra or Tocilizumab Significantly Decreased the Tested Parameters

**Figure 2:**
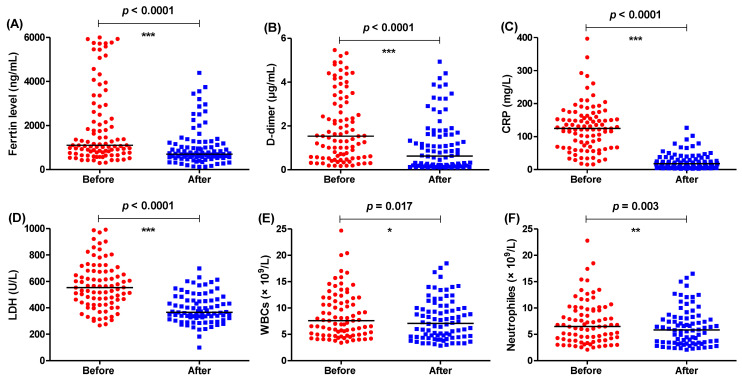
The levels of different parameters before and after administration of treatment regimen with interleukin-1 antagonist anakinra. The levels of (**A**) ferritin, (**B**) D-dimer, (**C**) CRP, (**D**) LDH, (**E**) WBCs, and (**F**) neutrophiles were measured before and after completion of treatment regimen. *** = *p* ≤ 0.001, ** = *p* < 0.01, * = *p* ≤ 0.05, and ns (nonsignificant) *p* > 0.05.

**Figure 3:**
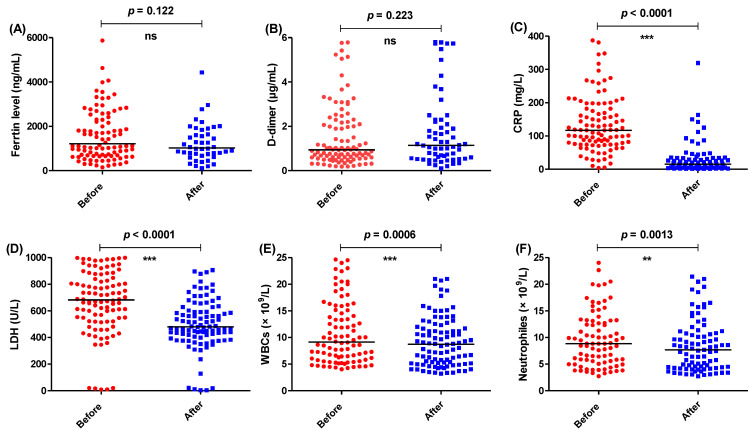
The levels of different parameters before and after the administration of the treatment regimen with interleukin-6 antagonist tocilizumab. The levels of (**A**) ferritin, (**B**) D-dimer, (**C**) CRP, (**D**) LDH, (**E**) WBCs, and (**F**) neutrophiles were measured before and after completion of treatment regimen. *** = *p* ≤ 0.001, ** = *p* < 0.01, and ns (nonsignificant) *p* > 0.05.

The tested parameters were measured before and after the administration of the same protocol but provided additionally with IL-1 antagonist anakinra (Figure 2) or IL-6 antagonist tocilizumab (Figure 3). Anakinra or tocilizumab diminished CRP, LDH, WBCs, and neutrophils after the accomplishment of the treatment as indicated in Table 1. Interestingly, the levels of ferritin and D-dimer were significantly decreased in the group treated with anakinra; in contrast, the levels were not significantly influenced with the treatment regimen including tocilizumab.

### 3.3. Anakinra Improved the Ferritin and D-Dimer Levels in Comparison to Tocilizumab and the Usual Treatment Regimen

**Figure 4:**
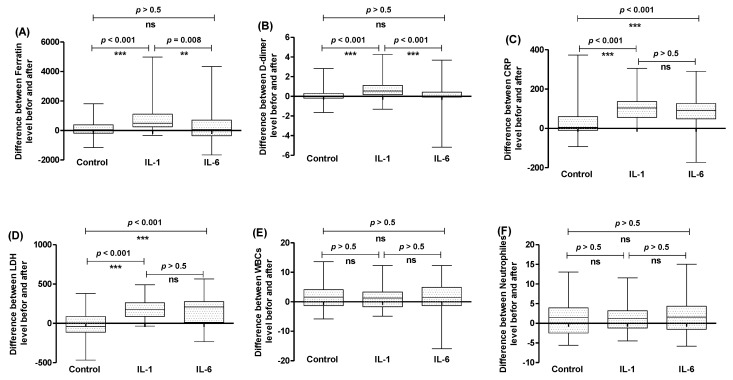
Comparison between the change in different parameter levels before and after administration of the treatment regimen without interleukin antagonists, or treatment regimens including IL−1 or IL−6 antagonists. The differences in levels of (**A**) ferritin, (**B**) D-dimer, (**C**) CRP, (**D**) LDH, (**E**) WBCs, and (**F**) neutrophiles were compared between the three treatment regimen. *** = *p* ≤ 0.001, ** = *p* < 0.01, and ns (nonsignificant) *p* > 0.05.

To compare between the effectiveness of the three tested treatment regimens, the differences between the measured values of each parameter before and after treatment were calculated, and then statistically evaluated. For ferritin and D-dimer levels, the differences between before and after values were significantly increased in the case of anakinra in comparison to the tocilizumab or control (usual) treatment regimen groups, indicating that anakinra significantly diminished the ferritin and D-dimer levels and improved their outcome in comparison to the tocilizumab or control groups (Figure 4A,B). Both anakinra and tocilizumab significantly improved the CRP and LDH levels in comparison to the usual treatment regimen; however, there were no significant differences between the effects of anakinra and tocilizumab on CRP and LDH (Figure 4C,D). There was no significant effect of anakinra or tocilizumab on the reduction of WBCs or neutrophils when compared with the usual treatment regimen (Figure 4E,F).

### 3.4. Anakinra Improved the Illness Outcome

**Figure 5:**
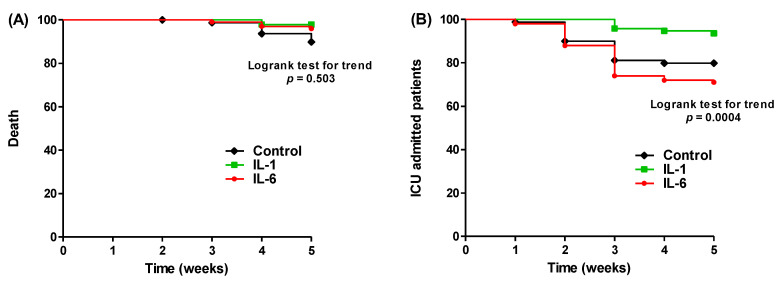
Kaplan–Meier plotting of (**A**) the death among patients after treatment with regimens including IL-1, IL-6 antagonists or not, (**B**) patients that were transferred to intensive care unit (ICU).

The treated patients in the three tested groups were observed for the next 5 weeks after completion of the treatment regimens as indicated in Table 1, and the patients who died or were transferred to the ICU were counted. The ICU-admitted patients were 16 out of 80 (20%), 6 out of 95 (6.3%), and 29 out of 100 (29%) in the usual treatment regimen, anakinra-containing regimen or tocilizumab-containing regimen, respectively. As can be seen, the IL-1 antagonist decreased the numbers of ICU-admitted patients from 20% (control group) and 29% (IL-6 antagonist group) to 6.3%. Furthermore, 8, 2, and 4 deaths were documented among the usual treatment regimen, anakinra containing regimen or tocilizumab containing regimen, respectively. In other words, the IL-1 antagonist decreased the death rate to 2.1% as compared to the IL-6 group (4%) or control group (10%). The numbers and dates of ICU admission or death were plotted using a Kaplan–Meier survival curve and the log rank test for trend was used to assess the statistical significance. There was no significant difference in survival time between the three treatment regimens (log rank test *p* = 0.503); however, there was a significant difference in the time to the ICU admission between the tested groups (log rank test *p* = 0.0004) (Figure 5).

A chi-square test followed by a Bonferroni post-hoc test was used to compare the difference in the proportion of numbers of deaths and ICU-admitted patients among the three tested groups. The chi-square test revealed a significant association between treatment regimens and ICU-admitted patients ($\chi^{2}{}_{\left(2\right)}$ = 8.075, *p* < 0.05). Post-hoc analyses using pairwise comparisons showed that the proportion of ICU-admitted patients in the group treated with anakinra was significantly lower than in the group treated with the usual regimen ($\chi^{2}{}_{\left(1\right)}$ = 7.019, *p* < 0.01) and in tocilizumab treated patients ($\chi^{2}{}_{\left(1\right)}$ = 5.714, *p* < 0.05). These findings indicate the beneficial effect of anakinra on the COVID-19 mild to moderate cases when administrated in the early stages after infection. On the other hand, there was no significant difference in the proportion of deaths recorded in the three treatment regimens ($\chi^{2}{}_{\left(2\right)}$ = 4.23, *p* = 0.12).

## 4. Discussion

The treatment regimens for COVID-19 have evolved over the course of the pandemic as more is learned about the virus and its pathogenesis. Unfortunately, the mild and moderate infections can become more severe and hospitalization may be necessary. Treatment should then include therapies that target the virus or the immune response to the virus [10,38]. The severity of the infection could be due mainly to the immune response overactivation that in turn results in a cytokine storm syndrome, which can be life-threatening [3,40]. The COVID-19 pandemic has highlighted the importance of immunomodulatory medications such as corticosteroids, IL-6 inhibitors, or IL-1 receptor antagonists, in dampening the immune response and reducing inflammation [32,40]. IL-1 and IL-6 are two key proinflammatory cytokines that are involved in the immune response to COVID-19 [18,20,26,35]. IL-1 antagonists, such as anakinra, and IL-6 inhibitors, such as tocilizumab, have been used to treat cytokine storm syndrome in COVID-19 patients [32,40]. The current study is aimed at evaluating the role of IL-1 or IL-6 antagonists in controlling the worsening of mild and moderate COVID-19 cases and preventing them from becoming severe.

Ferritin is primarily responsible for storing and releasing iron, which is essential for the growth and survival of many types of immune cells, including lymphocytes, macrophages, and neutrophils [60,61]. In the context of COVID-19, elevated levels of ferritin have been associated with severe disease and poor clinical outcomes [62,63,64]. In patients with COVID-19, elevated ferritin levels may reflect a hyperinflammation and cytokine storm syndrome [63,64]. While elevated ferritin levels may suggest the presence of severe disease, they are not specific to COVID-19 and can also be seen in other infections, autoimmune diseases, and malignancies [65]. In this study, patients who suffered from autoimmune diseases and cancers were excluded, and the influences on ferritin levels are due to the effect of the treatment regimens. However, there is no conclusive evidence to correlate the use of cortisones or IL-antagonists with ferritin levels in COVID-19. The effect on ferritin levels may depend on factors such as disease severity, treatment timing, and individual patient characteristics. Several studies have documented dexamethasone’s reducing effects on the ferritin levels of both critically ill and non-critically ill patients [66,67]. A few studies have reported that treatment with IL-6 antagonist tocilizumab could lead to a decrease in ferritin levels in severe COVID-19 cases [68,69]. On the other hand, there are other studies which showed the non-significant effect of dexamethasone [70,71,72] or IL-6 antagonist tocilizumab [73] on the patients who did not need ventilation or oxygen. This corresponds with our findings where dexamethasone and tocilizumab did not significantly reduce the ferritin levels in mild and moderate cases. Several studies have reported that treatment with anakinra can lead to a rapid decrease in ferritin levels in COVID-19 patients especially those with cytokine storm syndrome [74,75]. The current data show a significant decrease in ferritin levels after administration of anakinra. The exact mechanism behind this phenomenon is not well understood, but it may be related to the inhibition of IL-1 and other cytokines that drive the hyperinflammatory response in COVID-19 [18,28].

D-dimer levels have been found to be elevated in many patients with COVID-19, especially in those who develop severe disease [76]. This is thought to be due to the increased risk of blood clots that can occur in COVID-19, particularly in those patients having pre-existing conditions such as obesity, hypertension, and diabetes [77]. Elevated D-dimer levels have been identified as a predictor of disease severity and poor outcomes in patients with COVID-19 [77]. Studies have shown that the use of dexamethasone in COVID-19 patients can lead to a reduction in D-dimer levels [76,77]. While some studies have suggested that dexamethasone can reduce D-dimer levels in COVID-19 patients [78,79], other studies have found no significant effect [80,81,82]. While there have been some studies that have shown a reduction in D-dimer levels with the use of IL-6 antagonists in COVID-19 patients, other studies have found no significant effect on D-dimer levels. In a study carried out on 432 patients, for instance, tocilizumab led to a rapid and sustained reduction in D-dimer levels in hospitalized severe COVID-19 cases [83]. However, a randomized controlled study on 389 patients with moderate to severe COVID-19 documented that treatment with tocilizumab did not reduce the composite endpoint of death or the need for mechanical ventilation and did not significantly affect D-dimer levels compared to placebo [84]. Another study performed on 149 COVID-19 patients found that treatment with another IL-6 antagonist sarilumab did not significantly affect D-dimer levels in hospitalized COVID-19 patients compared to placebo [85]. In the present study, both tocilizumab and dexamethasone did not significantly decrease D-dimer levels in the mild and moderate cases. In contrast, anakinra significantly reduced the D-dimer levels in the studied cases, which is in agreement with other studies’ findings [86,87,88]. It is worth mentioning that anakinra significantly decreased the levels of ferritin and D-dimer when compared to tocilizumab.

LDH is an enzyme involved in a wide range of metabolic processes in the body, and is produced by the virus-infected cells as a result of the cellular damage that occurs during the infection. This release of LDH can act as a danger signal, alerting the immune system to the presence of the infection and triggering a response by production of cytokines such as IL-1 and IL-18, which can help to activate immune cells and fight off viral infections [89,90]. LDH is one of the biomarkers that is frequently measured in COVID-19 patients as its level is elevated due to damage of lung tissues during the infection [91]. In the current study, anakinra significantly diminished the LDH levels; in contrast, tocilizumab and dexamethasone did not show any significant effect. IL-1 promotion having an influence on the release of LDH from cells [92,93] could explain the IL-1 antagonist anakinra’s effect on decreasing LDH levels. This is consistent with other studies which showed the reducing effect of an IL-1 antagonist on LDH levels [74,88,94]. There is some conflicting evidence regarding the effects of dexamethasone and IL-6 antagonist on LDH levels in COVID-19. Some studies have suggested that dexamethasone may be associated with a decrease in LDH levels in COVID-19 patients [95,96]. There is some evidence to suggest that tocilizumab could reduce LDH levels in COVID-19 patients [97,98]. On the other hand, other studies have found no significant effect of dexamethasone or tocilizumab on LDH levels in COVID-19 patients [99,100].

In the current study, dexamethasone, tocilizumab, and anakinra significantly decreased the levels of CRP and levels of WBCs, which is in agreement with several published studies [69,101,102]. Previous studies suggested that prompt treatment of COVID-19 before intubation may be more important than the specific type of anti-inflammatory treatment. The present findings showed the significant influence of anakinra on the time to ICU admission and on decreasing the number of ICU-admitted patients as compared to tocilizumab or the usual regimen containing dexamethasone alone. There are several strengths of the current study including the establishment of the relationship between use of anakinra or tocilizumab and different biochemical markers such as ferritin, LDH, D-dimer, and CRP in mild to moderate COVID-19 cases. Furthermore, this prospective study allows for the collection of detailed information which increases the accuracy and specificity of findings; this in turn can provide valuable data for designing effective health interventions and informing public health policies. On the other hand, there were some limitations. The current study could be expanded by measuring more biochemical and clinical parameters, while increasing the number of participants in each group could strengthen the findings of this study. These findings demonstrate the effectiveness of anakinra in controlling the biochemical and hematological parameters and decreasing the progression of mild or moderate COVID-19 cases.

## 5. Conclusions

There are several treatments currently available for COVID-19, and the specific treatment options depend on the severity of the disease and other individual factors. The worst-case scenario is the prognosis of mild or moderate COVID-19 becoming severe and mandating ICU admission. The current study aimed to evaluate the use of IL-1 antagonist anakinra and IL-6 antagonist tocilizumab as well as corticosteroids in controlling mild or moderate COVID-19 cases. The present findings revealed a significant ability of anakinra to diminish several biomarkers such as ferritin, D-dimer, LDH, CRP and WBCs in COVID-19 patients in comparison to tocilizumab. Furthermore, anakinra decreased the number of ICU-admitted patients. This paves the way for clinicians to try using anakinra in controlling early stage COVID-19 and decreasing the need for ICU admission and mechanical ventilation.

## Figures and Tables

**Table 1:** Treatment regimens.

Usual Treatment Regimen (Control)	Treatment Regimen Including IL-1 Antagonist (IL-1)	Treatment Regimen Including IL-6 Antagonist (IL-6)
- Ceftriaxone 2 g every 24 h/5 days - Heparin low mol wt 4000 IU every 24 h/5 days - Calcium with vitamin D tablet 600 mg every 12 h/5 days - Paracetamol injection 1 g every 6 h/5 days - Butamirate citrate cough syrup 10 mL every 8 h/5 days - Dexamethasone injection 6 mg every 24 h/5 days	- Ceftriaxone 2 g every 24 h/5 days - Heparin low mol wt 4000 IU every 24 h/5 days - Calcium with vitamin D tablet 600 mg every 12 h/5 days - Paracetamol injection 1 g every 6 h/5 days - Butamirate citrate cough syrup 10 mL every 8 h/5 days - Dexamethasone injection 6 mg every 24 h/5 days - Anakinra injection 100 mg every 12 h/3 days. If no clinical improvement Anakinra injection 100 mg every 24 h/7 days.	- Ceftriaxone 2 g every 24 h/5 days - Heparin low mol wt 4000 IU every 24 h/5 days - Calcium with vitamin D tablet 600 mg every 12 h/5 days - Paracetamol injection 1 g every 6 h/5 days - Butamirate citrate cough syrup 10 mL every 8 h/5 days - Dexamethasone injection 6 mg every 24 h/5 days. - Tocilizumab injection dose 4–8 mg/kg every 24 h (max 800 mg/dose). - One additional dose may be considered 12 h later if clinical symptoms worsen or there is no clinical improvement.

**Table 2:** Demographic characteristics and clinical features of the cases.

Parameter	Did not Receive IL-Antagonists (Control)	Received IL-1 Antagonist (Anakinra)	Received IL-6 Antagonist (Tocilizumab)
Number	80	95	100
Age-median year (range)	44 (17–67)	54 (31–81)	53 (31–85)
Gender-no. (%)			
Male	65 (81.2)	56 (58.9)	84 (84)
Female	15 (18.8)	39 (41.1)	16 (16)
Liver Functions-median U/L (range)			
AST	37.8 (20–68)	33.7 (18–65)	38.1 (23–71)
ALT	39.2 (18–59)	40 (20–56)	45.4 (18–60)
Kidney Functions- median mg/dL (range)			
Creatinine	1.31 (0.86–2.1)	1.35 (0.5–2.2)	1.24 (0.64–2)
Body Mass Index (BMI)-range	19–38	18–44	20–35

## Data Availability

Data supporting this study are included within the article.

## References

[1] V’Kovski P., Kratzel A., Steiner S., Stalder H., Thiel V. (2021). Coronavirus biology and replication: Implications for SARS-CoV-2. Nat. Rev. Microbiol..

[2] Yang H., Rao Z. (2021). Structural biology of SARS-CoV-2 and implications for therapeutic development. Nat. Rev. Microbiol..

[3] Zhang H.P., Sun Y.L., Wang Y.F., Yazici D., Azkur D., Ogulur I., Azkur A.K., Yang Z.W., Chen X.X., Zhang A.Z. (2023). Recent developments in the immunopathology of COVID-19. Allergy.

[4] Cao Z., Gao W., Bao H., Feng H., Mei S., Chen P., Gao Y., Cui Z., Zhang Q., Meng X. (2023). VV116 versus Nirmatrelvir-Ritonavir for Oral Treatment of COVID-19. N. Engl. J. Med..

[5] Muralidar S., Ambi S.V., Sekaran S., Krishnan U.M. (2020). The emergence of COVID-19 as a global pandemic: Understanding the epidemiology, immune response and potential therapeutic targets of SARS-CoV-2. Biochimie.

[6] Jackson C.B., Farzan M., Chen B., Choe H. (2022). Mechanisms of SARS-CoV-2 entry into cells. Nat. Rev. Mol. Cell Biol..

[7] Kirtipal N., Bharadwaj S., Kang S.G. (2020). From SARS to SARS-CoV-2, insights on structure, pathogenicity and immunity aspects of pandemic human coronaviruses. Infect. Genet. Evol..

[8] Jayaweera M., Perera H., Gunawardana B., Manatunge J. (2020). Transmission of COVID-19 virus by droplets and aerosols: A critical review on the unresolved dichotomy. Environ. Res..

[9] Botta M., Caritg O., van Meenen D.M.P., Pacheco A., Tsonas A.M., Mooij W.E., Burgener A., Manrique Hehl T., Shrestha G.S., Horn J. (2023). Oxygen Consumption with High-Flow Nasal Oxygen versus Mechanical Ventilation- An International Multicenter Observational Study in COVID-19 Patients (PROXY-COVID). Am. J. Trop. Med. Hyg..

[10] Forchette L., Sebastian W., Liu T. (2021). A Comprehensive Review of COVID-19 Virology, Vaccines, Variants, and Therapeutics. Curr. Med. Sci..

[11] Svoboda J., Tkadlec J., Pavlogiannis A., Chatterjee K., Nowak M.A. (2022). Infection dynamics of COVID-19 virus under lockdown and reopening. Sci. Rep..

[12] Rivera L.C., Mohamed S., Salar T., Mostafa M.R., Najim M., Malik M.A., Renjithal S.L.M., Magdi M. (2023). Organizing Pneumonia: An Unusual Sequela of COVID-19 Infection. Eur. J. Case Rep. Intern. Med..

[13] Maliki I., Elmsellem H., Hafez B., EL Moussaoui A., Reda Kachmar M., Ouahbi A. (2021). The psychological properties of the Arabic BDI-II and the psychological state of the general Moroccan population during the mandatory quarantine due to the COVID-19 pandemic. Casp. J. Environ. Sci..

[14] Ahmad M.F., Mahakkanukrauh P., Das S. (2022). The Detection of Severe Acute Respiratory Syndrome Coronavirus 2 (SARS-CoV-2) Virus in the Vaginal Fluid of Females With Severe Coronavirus Disease 2019 (COVID-19) Infection: Scientific Facts. Clin. Infect. Dis..

[15] Chong Y.M., Chan Y.F., Jamaluddin M.F.H., Hasan M.S., Pang Y.K., Ponnampalavanar S., Syed Omar S.F., Sam I.C. (2022). Rhinovirus/enterovirus was the most common respiratory virus detected in adults with severe acute respiratory infections pre-COVID-19 in Kuala Lumpur, Malaysia. PLoS ONE.

[16] Mrcela D., Markic J., Zhao C., Viskovic D.V., Milic P., Copac R., Li Y. (2022). Changes following the Onset of the COVID-19 Pandemic in the Burden of Hospitalization for Respiratory Syncytial Virus Acute Lower Respiratory Infection in Children under Two Years: A Retrospective Study from Croatia. Viruses.

[17] Ali M.A.O., Abdalrahman N.A., Shanab E.A.I., Mohammed M.M.A., Ibrahim M.M., Abdalrahman I.B. (2023). The outcome of COVID-19 patients in the intensive care unit in Sudan: A cross-sectional study. Health Sci. Rep..

[18] Hu B., Huang S., Yin L. (2021). The cytokine storm and COVID-19. J. Med. Virol..

[19] Nile S.H., Nile A., Qiu J., Li L., Jia X., Kai G. (2020). COVID-19: Pathogenesis, cytokine storm and therapeutic potential of interferons. Cytokine Growth Factor Rev..

[20] Soy M., Keser G., Atagunduz P. (2021). Pathogenesis and treatment of cytokine storm in COVID-19. Turk. J. Biol..

[21] Ye Q., Wang B., Mao J. (2020). The pathogenesis and treatment of the; Cytokine Storm’ in COVID-19. J. Infect..

[22] Yongzhi X. (2021). COVID-19-associated cytokine storm syndrome and diagnostic principles: An old and new Issue. Emerg. Microbes Infect..

[23] Aldawsari M.F., Alalaiwe A., Khafagy E.S., Al Saqr A., Alshahrani S.M., Alsulays B.B., Alshehri S., Abu Lila A.S., Danish Rizvi S.M., Hegazy W.A.H. (2021). Efficacy of SPG-ODN 1826 Nanovehicles in Inducing M1 Phenotype through TLR-9 Activation in Murine Alveolar J774A.1 Cells: Plausible Nano-Immunotherapy for Lung Carcinoma. Int. J. Mol. Sci..

[24] Khayyat A.N., Abbas H.A., Mohamed M.F.A., Asfour H.Z., Khayat M.T., Ibrahim T.S., Youns M., Khafagy E.-S., Abu Lila A.S., Safo M.K. (2021). Not Only Antimicrobial: Metronidazole Mitigates the Virulence of Proteus mirabilis Isolated from Macerated Diabetic Foot Ulcer. Appl. Sci..

[25] Askoura M., Almalki A.J., Lila A.S.A., Almansour K., Alshammari F., Khafagy E.-S., Ibrahim T.S., Hegazy W.A.H. (2021). Alteration of Salmonella enterica Virulence and Host Pathogenesis through Targeting sdiA by Using the CRISPR-Cas9 System. Microorganisms.

[26] Ramasamy S., Subbian S. (2021). Critical Determinants of Cytokine Storm and Type I Interferon Response in COVID-19 Pathogenesis. Clin. Microbiol. Rev..

[27] Soy M., Keser G., Atagunduz P., Tabak F., Atagunduz I., Kayhan S. (2020). Cytokine storm in COVID-19: Pathogenesis and overview of anti-inflammatory agents used in treatment. Clin. Rheumatol..

[28] Fara A., Mitrev Z., Rosalia R.A., Assas B.M. (2020). Cytokine storm and COVID-19: A chronicle of pro-inflammatory cytokines. Open Biol..

[29] Hu Z., Li S., Song X. (2021). Cytokine storm with rapidly elevated interleukin-6 indicates sudden death in patients with critical COVID-19. Cytokine Growth Factor Rev..

[30] Conti P., Caraffa A., Tete G., Gallenga C.E., Ross R., Kritas S.K., Frydas I., Younes A., Di Emidio P., Ronconi G. (2020). Mast cells activated by SARS-CoV-2 release histamine which increases IL-1 levels causing cytokine storm and inflammatory reaction in COVID-19. J. Biol. Regul. Homeost. Agents.

[31] Jafrin S., Aziz M.A., Islam M.S. (2022). Elevated Levels of Pleiotropic Interleukin-6 (IL-6) and Interleukin-10 (IL-10) are Critically Involved With the Severity and Mortality of COVID-19: An Updated Longitudinal Meta-Analysis and Systematic Review on 147 Studies. Biomark. Insights.

[32] Mojtabavi H., Saghazadeh A., Rezaei N. (2020). Interleukin-6 and severe COVID-19: A systematic review and meta-analysis. Eur. Cytokine Netw..

[33] Kitsos D., Tzartos J., Korres G., Giannopapas V., Riga M., Stergiou C., Tsoga A., Grigoropoulos C., Paraskevas G., Zompola C. (2023). IL-6 Serum Levels in COVID-19 Patients With Vertigo. Cureus.

[34] Sasson J., Moreau G.B., Petri W.A. (2023). The Role of IL-13 and the Type 2 Immune Pathway in COVID-19: A Review. Ann. Allergy Asthma Immunol..

[35] Pinzon R.T., Wijaya V.O., Buana R.B. (2021). Interleukin-6 (IL-6) inhibitors as therapeutic agents for coronavirus disease 2019 (COVID-19): A systematic review and meta-analysis. J. Infect. Public Health.

[36] Udomsinprasert W., Jittikoon J., Sangroongruangsri S., Chaikledkaew U. (2021). Circulating Levels of Interleukin-6 and Interleukin-10, But Not Tumor Necrosis Factor-Alpha, as Potential Biomarkers of Severity and Mortality for COVID-19: Systematic Review with Meta-analysis. J. Clin. Immunol..

[37] Yu S.Y., Koh D.H., Choi M., Ryoo S., Huh K., Yeom J.S., Yoon Y.K. (2022). Clinical efficacy and safety of interleukin-6 receptor antagonists (tocilizumab and sarilumab) in patients with COVID-19: A systematic review and meta-analysis. Emerg. Microbes Infect..

[38] Conti P., Caraffa A., Gallenga C.E., Ross R., Kritas S.K., Frydas I., Younes A., Ronconi G. (2020). Coronavirus-19 (SARS-CoV-2) induces acute severe lung inflammation via IL-1 causing cytokine storm in COVID-19: A promising inhibitory strategy. J. Biol. Regul. Homeost. Agents.

[39] Kim J.S., Lee J.Y., Yang J.W., Lee K.H., Effenberger M., Szpirt W., Kronbichler A., Shin J.I. (2021). Immunopathogenesis and treatment of cytokine storm in COVID-19. Theranostics.

[40] Kaps L., Labenz C., Grimm D., Schwarting A., Galle P.R., Schreiner O. (2020). Treatment of cytokine storm syndrome with IL-1 receptor antagonist anakinra in a patient with ARDS caused by COVID-19 infection: A case report. Clin. Case Rep..

[41] Askoura M., Abbas H.A., Al Sadoun H., Abdulaal W.H., Abu Lila A.S., Almansour K., Alshammari F., Khafagy E.-S., Ibrahim T.S., Hegazy W.A.H. (2022). Elevated Levels of IL-33, IL-17 and IL-25 Indicate the Progression from Chronicity to Hepatocellular Carcinoma in Hepatitis C Virus Patients. Pathogens.

[42] Conti P., Ronconi G., Caraffa A., Gallenga C.E., Ross R., Frydas I., Kritas S.K. (2020). Induction of pro-inflammatory cytokines (IL-1 and IL-6) and lung inflammation by Coronavirus-19 (COVI-19 or SARS-CoV-2): Anti-inflammatory strategies. J. Biol. Regul. Homeost. Agents.

[43] Hegazy W.A.H., Henaway M. (2015). Hepatitis C virus pathogenesis: Serum IL-33 level indicates liver damage. Afr. J. Microbiol. Res..

[44] Rondovic G., Djordjevic D., Udovicic I., Stanojevic I., Zeba S., Abazovic T., Vojvodic D., Abazovic D., Khan W., Surbatovic M. (2022). From Cytokine Storm to Cytokine Breeze: Did Lessons Learned from Immunopathogenesis Improve Immunomodulatory Treatment of Moderate-to-Severe COVID-19?. Biomedicines.

[45] Han Q., Guo M., Zheng Y., Zhang Y., De Y., Xu C., Zhang L., Sun R., Lv Y., Liang Y. (2020). Current Evidence of Interleukin-6 Signaling Inhibitors in Patients With COVID-19: A Systematic Review and Meta-Analysis. Front. Pharmacol..

[46] Bovet M., Wadsack D., Kosely F., Zink W., Zahn R. (2021). Fatal course of COVID-19 despite IL-6 receptor blockade in cytokine storm: Perimyocarditis and coagulopathy after administration of tocilizumab. Anaesthesist.

[47] Emsley H., Smith C., Georgiou R., Vail A., Hopkins S., Rothwell N., Tyrrell P. (2005). A randomised phase II study of interleukin-1 receptor antagonist in acute stroke patients. J. Neurol. Neurosurg. Psychiatry.

[48] Möller B., Villiger P.M. (2006). Inhibition of IL-1, IL-6, and TNF-α in immune-mediated inflammatory diseases. Springer Seminars in Immunopathology.

[49] Scherger S., Henao-Martínez A., Franco-Paredes C., Shapiro L. (2020). Rethinking interleukin-6 blockade for treatment of COVID-19. Med. Hypotheses.

[50] Atal S., Fatima Z. (2020). IL-6 inhibitors in the treatment of serious COVID-19: A promising therapy?. Pharm. Med..

[51] Khayyat A.N., Abbas H.A., Khayat M.T., Shaldam M.A., Askoura M., Asfour H.Z., Khafagy E.-S., Abu Lila A.S., Allam A.N., Hegazy W.A.H. (2021). Secnidazole Is a Promising Imidazole Mitigator of Serratia marcescens Virulence. Microorganisms.

[52] Bertoni A., Penco F., Mollica H., Bocca P., Prigione I., Corcione A., Cangelosi D., Schena F., Del Zotto G., Amaro A. (2022). Spontaneous NLRP3 inflammasome-driven IL-1-beta secretion is induced in severe COVID-19 patients and responds to anakinra treatment. J. Allergy Clin. Immunol..

[53] Cavalli G., Dagna L. (2021). The right place for IL-1 inhibition in COVID-19. Lancet Respir. Med..

[54] Della-Torre E., Criscuolo E., Lanzillotta M., Locatelli M., Clementi N., Mancini N., Dagna L., COVID-BioB study group (2021). IL-1 and IL-6 inhibition affects the neutralising activity of anti-SARS-CoV-2 antibodies in patients with COVID-19. Lancet Rheumatol..

[55] Della-Torre E., Lanzillotta M., Campochiaro C., Cavalli G., De Luca G., Tomelleri A., Boffini N., De Lorenzo R., Ruggeri A., Rovere-Querini P. (2021). Respiratory Impairment Predicts Response to IL-1 and IL-6 Blockade in COVID-19 Patients With Severe Pneumonia and Hyper-Inflammation. Front. Immunol..

[56] Franzetti M., Forastieri A., Borsa N., Pandolfo A., Molteni C., Borghesi L., Pontiggia S., Evasi G., Guiotto L., Erba M. (2021). IL-1 Receptor Antagonist Anakinra in the Treatment of COVID-19 Acute Respiratory Distress Syndrome: A Retrospective, Observational Study. J. Immunol..

[57] Makaremi S., Asgarzadeh A., Kianfar H., Mohammadnia A., Asghariazar V., Safarzadeh E. (2022). The role of IL-1 family of cytokines and receptors in pathogenesis of COVID-19. Inflamm. Res..

[58] Renieris G., Karakike E., Gkavogianni T., Droggiti D.E., Stylianakis E., Andriopoulou T., Spanou V.M., Kafousopoulos D., Netea M.G., Eugen-Olsen J. (2022). IL-1 Mediates Tissue-Specific Inflammation and Severe Respiratory Failure in COVID-19. J. Innate Immun..

[59] van de Veerdonk F.L., Netea M.G. (2020). Blocking IL-1 to prevent respiratory failure in COVID-19. Crit. Care.

[60] Cassat J.E., Skaar E.P. (2013). Iron in infection and immunity. Cell Host Microbe.

[61] Haschka D., Hoffmann A., Weiss G. (2021). Iron in immune cell function and host defense. Semin. Cell Dev. Biol..

[62] Alroomi M., Rajan R., Omar A.A., Alsaber A., Pan J., Fatemi M., Zhanna K.D., Aboelhassan W., Almutairi F., Alotaibi N. (2021). Ferritin level: A predictor of severity and mortality in hospitalized COVID-19 patients. Immun. Inflamm. Dis..

[63] Dahan S., Segal G., Katz I., Hellou T., Tietel M., Bryk G., Amital H., Shoenfeld Y., Dagan A. (2020). Ferritin as a marker of severity in COVID-19 patients: A fatal correlation. Isr. Med. Assoc. J. IMAJ.

[64] Lin Z., Long F., Yang Y., Chen X., Xu L., Yang M. (2020). Serum ferritin as an independent risk factor for severity in COVID-19 patients. J. Infect..

[65] Mahroum N., Alghory A., Kiyak Z., Alwani A., Seida R., Alrais M., Shoenfeld Y. (2022). Ferritin–from iron, through inflammation and autoimmunity, to COVID-19. J. Autoimmun..

[66] Dupuis C., de Montmollin E., Buetti N., Goldgran-Toledano D., Reignier J., Schwebel C., Domitile J., Neuville M., Ursino M., Siami S. (2021). Impact of early corticosteroids on 60-day mortality in critically ill patients with COVID-19: A multicenter cohort study of the OUTCOMEREA network. PLoS ONE.

[67] Moreno A., Vargas C., Azocar F., Villarroel F., Cofré M., Oppliger H., Ríos F., Raijmakers M., Silva-Ayarza I., Beltrán C. (2021). Steroids and mortality in non-critically ill COVID-19 patients: A propensity score-weighted study in a Chilean cohort. Int. J. Infect. Dis..

[68] Corominas H., Castellví I., Pomar V., Antonijoan R., Mur I., Matas L., Gich I., de Benito N., Laiz A., Castillo D. (2021). Effectiveness and safety of intravenous tocilizumab to treat COVID-19-associated hyperinflammatory syndrome: Covizumab-6 observational cohort. Clin. Immunol..

[69] Hashimoto S., Yoshizaki K., Uno K., Kitajima H., Arai T., Tamura Y., Morishita H., Matsuoka H., Han Y., Minamoto S. (2021). Prompt reduction in CRP, IL-6, IFN-γ, IP-10, and MCP-1 and a relatively low basal ratio of ferritin/CRP is possibly associated with the efficacy of tocilizumab Monotherapy in severely to critically ill patients with COVID-19. Front. Med..

[70] Ahmed M.H., Hassan A. (2020). Dexamethasone for the treatment of coronavirus disease (COVID-19): A review. SN Compr. Clin. Med..

[71] Horby P., Lim W.S., Emberson J., Mafham M., Bell J., Linsell L., Staplin N., Brightling C., Ustianowski A., Elmahi E. (2020). Effect of dexamethasone in hospitalized patients with COVID-19–preliminary report. MedRxiv.

[72] Albani F., Fusina F., Granato E., Capotosto C., Ceracchi C., Gargaruti R., Santangelo G., Schiavone L., Taranto M.S., Tosati C. (2021). Corticosteroid treatment has no effect on hospital mortality in COVID-19 patients. Sci. Rep..

[73] Guz D., Gafter-Gvili A., Lev N., Levin G.S., Lev S. (2022). Tocilizumab Treatment Effect on Iron Homeostasis in Severe COVID-19 Patients. Acta Haematol..

[74] Navarro-Millán I., Sattui S.E., Lakhanpal A., Zisa D., Siegel C.H., Crow M.K. (2020). Use of anakinra to prevent mechanical ventilation in severe COVID-19: A case series. Arthritis Rheumatol..

[75] Kyriazopoulou E., Huet T., Cavalli G., Gori A., Kyprianou M., Pickkers P., Eugen-Olsen J., Clerici M., Veas F., Chatellier G. (2021). Effect of anakinra on mortality in patients with COVID-19: A systematic review and patient-level meta-analysis. Lancet Rheumatol..

[76] Rostami M., Mansouritorghabeh H. (2020). D-dimer level in COVID-19 infection: A systematic review. Expert Rev. Hematol..

[77] Alzoughool F., Alanagreh L.a., Abumweis S., Atoum M. (2021). Cerebrovascular comorbidity, high blood levels of C-reactive protein and D-dimer are associated with disease outcomes in COVID-19 patients. Clin. Hemorheol. Microcirc..

[78] Takahashi H., Iwasaki Y., Watanabe T., Ichinose N., Oda T. (2022). Pulmonary embolism after dexamethasone treatment for COVID-19: A case report. BMC Infect. Dis..

[79] Wenban C., Heer R.S., Baktash V., Kandiah P., Katsanouli T., Pandey A., Goindoo R., Ajaz A., Van den Abbeele K., Mandal A.K. (2021). Dexamethasone treatment may mitigate adverse effects of vitamin D deficiency in hospitalized COVID-19 patients. J. Med. Virol..

[80] Sarfraz A., Sarfraz Z., Razzack A.A., Patel G., Sarfraz M. (2021). Venous thromboembolism, corticosteroids and COVID-19: A systematic review and meta-analysis. Clin. Appl. Thromb./Hemost..

[81] Lu C., Liu Y., Chen B., Yang H., Hu H., Liu Y., Zhao Y. (2021). Prognostic value of lymphocyte count in severe COVID-19 patients with corticosteroid treatment. Signal Transduct. Target. Ther..

[82] Marrone A., Nevola R., Sellitto A., Cozzolino D., Romano C., Cuomo G., Aprea C., Schwartzbaum M.X.P., Ricozzi C., Imbriani S. (2022). Remdesivir plus dexamethasone versus dexamethasone alone for the treatment of COVID-19 patients requiring supplemental O2 therapy: A prospective controlled non-randomized study. Clin. Infect. Dis..

[83] Ip A., Berry D.A., Hansen E., Goy A.H., Pecora A.L., Sinclaire B.A., Bednarz U., Marafelias M., Berry S.M., Berry N.S. (2020). Hydroxychloroquine and tocilizumab therapy in COVID-19 patients—An observational study. PLoS ONE.

[84] Salama C., Han J., Yau L., Reiss W.G., Kramer B., Neidhart J.D., Criner G.J., Kaplan-Lewis E., Baden R., Pandit L. (2021). Tocilizumab in Patients Hospitalized with COVID-19 Pneumonia. N. Engl. J. Med..

[85] Mariette X., Hermine O., Tharaux P.-L., Resche-Rigon M., Porcher R., Ravaud P., Bureau S., Dougados M., Tibi A., Azoulay E. (2022). Sarilumab in adults hospitalised with moderate-to-severe COVID-19 pneumonia (CORIMUNO-SARI-1): An open-label randomised controlled trial. Lancet Rheumatol..

[86] Balkhair A., Al-Zakwani I., Al Busaidi M., Al-Khirbash A., Al Mubaihsi S., BaTaher H., Al Aghbari J., Al Busaidi I., Al Kindi M., Baawain S. (2021). Anakinra in hospitalized patients with severe COVID-19 pneumonia requiring oxygen therapy: Results of a prospective, open-label, interventional study. Int. J. Infect. Dis..

[87] Naveed Z., Sarwar M., Ali Z., Saeed D., Choudhry K., Sarfraz A., Sarfraz Z., Felix M., Cherrez-Ojeda I. (2022). Anakinra treatment efficacy in reduction of inflammatory biomarkers in COVID-19 patients: A meta-analysis. J. Clin. Lab. Anal..

[88] Khani E., Shahrabi M., Rezaei H., Pourkarim F., Afsharirad H., Solduzian M. (2022). Current evidence on the use of anakinra in COVID-19. Int. Immunopharmacol..

[89] Ding J., Karp J.E., Emadi A. (2017). Elevated lactate dehydrogenase (LDH) can be a marker of immune suppression in cancer: Interplay between hematologic and solid neoplastic clones and their microenvironments. Cancer Biomark..

[90] Robertson S.J., Ammann C.G., Messer R.J., Carmody A.B., Myers L., Dittmer U., Nair S., Gerlach N., Evans L.H., Cafruny W.A. (2008). Suppression of acute anti-friend virus CD8+ T-cell responses by coinfection with lactate dehydrogenase-elevating virus. J. Virol..

[91] Henry B.M., Aggarwal G., Wong J., Benoit S., Vikse J., Plebani M., Lippi G. (2020). Lactate dehydrogenase levels predict coronavirus disease 2019 (COVID-19) severity and mortality: A pooled analysis. Am. J. Emerg. Med..

[92] Levine S.J., Wu T., Shelhamer J.H. (1997). Extracellular release of the type I intracellular IL-1 receptor antagonist from human airway epithelial cells: Differential effects of IL-4, IL-13, IFN-gamma, and corticosteroids. J. Immunol. (Baltim. Md. 1950).

[93] Perregaux D., Barberia J., Lanzetti A.J., Geoghegan K.F., Carty T., Gabel C. (1992). IL-1 beta maturation: Evidence that mature cytokine formation can be induced specifically by nigericin. J. Immunol. (Baltim. Md. 1950).

[94] Pontali E., Volpi S., Signori A., Antonucci G., Castellaneta M., Buzzi D., Montale A., Bustaffa M., Angelelli A., Caorsi R. (2021). Efficacy of early anti-inflammatory treatment with high doses of intravenous anakinra with or without glucocorticoids in patients with severe COVID-19 pneumonia. J. Allergy Clin. Immunol..

[95] Pinzón M.A., Ortiz S., Holguín H., Betancur J.F., Cardona Arango D., Laniado H., Arias Arias C., Muñoz B., Quiceno J., Jaramillo D. (2021). Dexamethasone vs methylprednisolone high dose for COVID-19 pneumonia. PLoS ONE.

[96] Yildirim F., Erdogan M., Mutlu M., Icacan O., Onar M., Bes C. (2022). Efficacy of anticytokine treatments added to corticosteroids in patients with COVID-19-associated pneumonia and hyperinflammation: A single center experience. Eur. Rev. Med. Pharmacol. Sci..

[97] Sarhan R.M., Harb H.S., Abou Warda A.E., Salem-Bekhit M.M., Shakeel F., Alzahrani S.A., Madney Y.M., Boshra M.S. (2022). Efficacy of the early treatment with tocilizumab-hydroxychloroquine and tocilizumab-remdesivir in severe COVID-19 Patients. J. Infect. Public Health.

[98] Fernández-Ruiz M., López-Medrano F., Pérez-Jacoiste Asín M.A., Maestro de la Calle G., Bueno H., Caro-Teller J.M., Catalan M., de la Calle C., García-García R., Gómez C. (2021). Tocilizumab for the treatment of adult patients with severe COVID-19 pneumonia: A single-center cohort study. J. Med. Virol..

[99] Zarębska-Michaluk D., Jaroszewicz J., Rogalska M., Martonik D., Pabjan P., Berkan-Kawińska A., Bolewska B., Oczko-Grzesik B., Kozielewicz D., Tudrujek-Zdunek M. (2021). Effectiveness of tocilizumab with and without dexamethasone in patients with severe COVID-19: A retrospective study. J. Inflamm. Res..

[100] Edalatifard M., Akhtari M., Salehi M., Naderi Z., Jamshidi A., Mostafaei S., Najafizadeh S.R., Farhadi E., Jalili N., Esfahani M. (2020). Intravenous methylprednisolone pulse as a treatment for hospitalised severe COVID-19 patients: Results from a randomised controlled clinical trial. Eur. Respir. J..

[101] Ajeganova S., De Becker A., Schots R. (2020). Efficacy of high-dose anakinra in refractory macrophage activation syndrome in adult-onset Still’s disease: When dosage matters in overcoming secondary therapy resistance. Ther. Adv. Musculoskelet. Dis..

[102] Kumakura S., Murakawa Y. (2014). Clinical characteristics and treatment outcomes of autoimmune-associated hemophagocytic syndrome in adults. Arthritis Rheumatol..

